# 734 A User-Centered Design Approach for a Burn Recovery Mobile Platform

**DOI:** 10.1093/jbcr/irad045.209

**Published:** 2023-05-15

**Authors:** Kyler Menge, Tam Pham, Gretchen Carrougher, Barclay Stewart, T Varugis Kurien

**Affiliations:** University of Washington, Tumwater, Washington; University of Washington, Seattle, Washington; University of Washington, Seattle, Washington; University of Washington, Seattle, Washington; University of Washington, Seattle, Washington; University of Washington, Tumwater, Washington; University of Washington, Seattle, Washington; University of Washington, Seattle, Washington; University of Washington, Seattle, Washington; University of Washington, Seattle, Washington; University of Washington, Tumwater, Washington; University of Washington, Seattle, Washington; University of Washington, Seattle, Washington; University of Washington, Seattle, Washington; University of Washington, Seattle, Washington; University of Washington, Tumwater, Washington; University of Washington, Seattle, Washington; University of Washington, Seattle, Washington; University of Washington, Seattle, Washington; University of Washington, Seattle, Washington; University of Washington, Tumwater, Washington; University of Washington, Seattle, Washington; University of Washington, Seattle, Washington; University of Washington, Seattle, Washington; University of Washington, Seattle, Washington

## Abstract

**Introduction:**

People with burn injuries experience challenges in multiple domains including wound and scar management, pain/pruritus, psychosocial recovery, return-to-work, and community re-integration. Mobile software platforms can provide clear, concise, and consumable information across different stakeholder groups. These platforms require a user experience (UX) that values users’ expressed needs to maximize engagement with critical information. This study seeks to inform the UX for an outpatient mobile software platform to optimize recovery.

**Methods:**

We performed semi-structured, in-depth interviews at a regional burn center with patients, clinicians, and carers to understand participant background, personal values, and requirements of a recovery platform. Interviews were transcribed and analyzed using grounded theory analysis and affinity mapping to surface major themes.

**Results:**

Fourteen in-depth interviews (over 13.5 hours) were conducted with six patients, six clinicians, and two carers. Thematic analysis identified four principal themes and corresponding UX requirements: (1) Provide a clear path to recovery: need for easily understood temporal visualizations of recovery experiences in multiple domains. This includes outlining paths that list relatively individualized tasks critical for recovery. (2) Facilitate task habituation: UX to ease successful task performance such as daily schedules, reminders and integration of recovery tasks with activities of daily living. (3) Maintain motivation: recovery progress tracking, motivational messages and visualization of long-term recovery trends to maximize task adherence. Patients and clinicians identified goal-authoring and recovery progress as key motivators in performing recovery tasks. (4) Create a burn community: access to other people with burn injuries through integrated forums. Neither patients nor clinicians desired to directly communicate with each other through the platform, instead preferring existing electronic health record methods.

**Conclusions:**

This study identified initial UX requirements for a person with burn injury-centered platform. There is strong support for temporal representations of recovery paths across multiple domains, UX design to facilitate and motivate users to stay engaged in performing critical recovery tasks, and access to the burn community.

**Applicability of Research to Practice:**

Interviews of prospective users outlined four important features to include in the UX design of an online burn recovery platform.

